# Characterisation of Fecal Soap Fatty Acids, Calcium Contents, Bacterial Community and Short-Chain Fatty Acids in Sprague Dawley Rats Fed with Different *sn*-2 Palmitic Triacylglycerols Diets

**DOI:** 10.1371/journal.pone.0164894

**Published:** 2016-10-26

**Authors:** Jianchun Wan, Songyou Hu, Kefeng Ni, Guifang Chang, Xiangjun Sun, Liangli Yu

**Affiliations:** 1 Institute of Food and Nutraceutical Science, School of Agriculture and Biology, Shanghai Jiao Tong University, Shanghai, PR China; 2 Wilmar (Shanghai) Biotechnology Research & Development Center Co., Ltd, Shanghai, PR China; 3 Department of Nutrition and Food Science, University of Maryland, College Park, MD, United States of America; University of Barcelona, Faculty of Biology, SPAIN

## Abstract

The structure of dietary triacylglycerols is thought to influence fatty acid and calcium absorption, as well as intestinal microbiota population of the host. In the present study, we investigated the impact of palmitic acid (PA) esterified at the *sn*-2 position on absorption of fatty acid and calcium and composition of intestinal microorganisms in rats fed high-fat diets containing either low *sn*-2 PA (12.1%), medium *sn*-2 PA (40.4%) or high *sn*-2 PA (56.3%), respectively. Fecal fatty acid profiles in the soaps were measured by gas chromatography (GC), while fecal calcium concentration was detected by ICP-MS. The fecal microbial composition was assessed using a 16S rRNA high-throughput sequencing technology and fecal short-chain fatty acids were detected by ion chromatograph. Dietary supplementation with a high *sn*-2 PA fat significantly reduced total fecal contents of fatty acids soap and calcium compared with the medium or low *sn*-2 PA fat groups. Diet supplementation with *sn*-2 PA fat did not change the entire profile of the gut microbiota community at phylum level and the difference at genera level also were minimal in the three treatment groups. However, high *sn*-2 PA fat diet could potentially improve total short-chain fatty acids content in the feces, suggesting that high dietary *sn*-2 PA fat might have a beneficial effect on host intestinal health.

## Introduction

Palmitic acid (PA) is one of the major fatty acids in breast milk fat (BMF) and accounts for 17–25% of the total fatty acids. Importantly, over 70% of PA is esterified at the *sn*-2 position in milk triacylglycerols [1]. In contrast to BMF, PA, the predominant saturated fatty acid in palm oil and other vegetable oil used in infant milk formulas is primarily esterified at the *sn*-1, 3 positions, whereas the *sn*-2 position is occupied by unsaturated fatty acids.

Recently, a structured triacylglycerol product with greater ratio of PA esterified at the *sn*-2 position was produced by enzymatic interesterification of palm stearine and oleic acid to mimic the structure of BMF [2]. It has been reported previously that a greater relative percentage of PA esterified at the *sn*-2 position was associated with enhanced fatty acid and calcium absorptions in rats and preterm infants [1, 3]. Conversely, Nelson et al. [4] reported that use of palm olein, containing only a very lower relative percentage of PA esterified at the *sn*-2 position, in the infant formulas reduced intestinal absorption of fat and calcium. These observations indicated that the position of PA in the oil of infant formula is important for the absorption of fat and calcium. Therefore, it is necessary to evaluate whether *sn*-2 PA levels may dose-dependently alter dietary fatty acid and calcium absorption. The intestinal microbiota have been recognized for their potential impact on the health of the host [5]. It has been shown that a disturbance in the gut microbial balance increased the risk of several human diseases or health problems, including but not being limited to obesity [6], malnutrition [7], diabetes [8], inflammatory bowel [9]and autoimmune diseases [10]. The intestinal microbial population can be modulated by specific dietary fatty acids, such as saturated fatty acids (SFA) [11, 12], monounsaturated fatty acids (MUFA) [13] and polyunsaturated fatty acids (PUFA) [14,15]. But little is known if the structure of triacylglycerols is important for altering the intestinal microbiota composition. Although previous studies demonstrated that high percentage of *sn*-2 PA structured triacylglycerols in infant formula promoted beneficial gut microbiota, such as *Lactobacillus* and *Bifidobacterium*, in stool compared with low percentage of *sn*-2 PA structured triacylglycerols using a culture-dependent technique [16] and fluorescent *in situ* hybridization (FISH) technique [17]. However, the conclusions are not representative for most bacterial species in the gut. In recent years, high-throughput next-generation sequencing has been widely used for profile gut microbiota composition and diversity [18]. Compared to the culture-dependent and FISH methods, the 16S rRNA high-throughput sequencing technology, together with the bioinformatics techniques can profile the microbial communities at high resolution and provide a profound insights into community structures. Therefore, it is interesting to further understand the influence of *sn*-2 PA structured triacylglycerols on the overall gut flora populations using 16S rRNA high-throughput sequencing technology.

In the present study, different percentage of PA esterified at the *sn*-2 position in dietary fat were investigated for their impact on absorptions of dietary fatty acids and calcium, intestinal microbiota composition and short-chain fatty acids (SCFA) production in Sprague Dawley (SD) rats using GC, and ICP-MS analyses,16S rRNA high-throughput sequencing technology and ion chromatography, respectively.

## Materials and Methods

### High*sn*-2 PA fat produced from shortening fat

Shortening fat and Palm olein (iodine value = 56)were gifts from Kerry Oils & Grains Industries Co., Ltd (Shanghai, China).The shortening fat was produced from blends of palm stearin (iodine value = 15) and high oleic sunflower oil (3:1 w/w) submitted to chemical interesterification using 0.2wt. % sodium methoxide as the catalyst and then the reactant was moved to bleaching and deodorization process. Oleic acid (OA7075, 78% C18:1) was obtained from Kerry Oleochemical Industrial Co., Ltd (Shanghai, China). Lipozyme RM IM was purchased from Novozymes (Tianjin, China).

The Lipozyme RM IM-catalyzed acidolysis between shortening fat and oleic acid was carried out in a pilot scale with a packed lipase bed reactor according to the methods reported by Xu et al. [19] with minor modifications. The lipase acidolysis conditions were as follows: column dimension was of 20 cm (i.d.) × 100 cm (length) and packed with 100 g Lipozyme RM IM, reaction temperature was 65°C, substrate ratio of shortening fat to oleic was 1:2 (w/w), and feeding rate was 150 g/h. Total of 30 kg reactants was produced. The free fatty acids in the reactants were removed by pilot molecular distillation (VTA125-20-SKR-G, Niederwinkling, Germany) and the distilled product was bleached and deodorized in a pilot oil refining facility. After deodorization, 8 kg of high *sn*-2 PA fat was obtained and stored at -20°C until further use.

### Fatty acid composition analysis

The determination of fatty acid composition was carried out using gas chromatograph with flame ionization detection (GC-FID) according to the IUPAC 2.301 and 2.302 official methods [20].

### *Sn*-2 positional fatty acids analysis

The fatty acids at the *sn*-2 position were determined in accordance with the AOCS Ch 3–91 official methods [21]. Briefly, the samples were hydrolyzed by pancreatic lipase to get 2-monoacylglycerols (MAG). The hydrolysis products were analyzed by preparative thin-layer chromatography (TLC) using the mobile phase n-hexane/diethyl ether/formic acid 70:30:1 (v/v/v).Analysis of the 2-MAG spot by GC gave the fatty acid profile of position 2 of triacylglycerols.

### Animals and diets

This study was conducted in conformity with the policies and procedures of the Institutional Animal Care and Use Committee of Shanghai University of Traditional Chinese Medicine (approved ethic number: SZY2016002). Thirty-six male SD rats (21 day-old, 61.66 ± 3.73 g) were purchased from Shanghai Experimental Animal Center of Chinese Academy of Sciences, and randomly assigned to a low *sn*-2 PA fat diet (low *sn*-2 PA fat: palm olein), medium *sn*-2 PA fat diet (Medium *sn*-2 PA fat: obtained by blending 30% weight of palm olein with 70% weight of *sn*-2 PA high fat), or a high *sn*-2 PA fat diet (high *sn*-2 PA fat) group. Animals were acclimatized to the environmentally standard condition (12 h light/darkness cycle, 22±2°C, 50–60% humidity) for 1 week prior to the introduction of experimental diets, which the animals consumed for 4 weeks. The water and feeds were offered *ad libitum*. The experimental diets were prepared by mixing commercial no fat diets (Research diets D04112303, New Brunswick, NJ, USA) with 15 wt. % experimental fats. The diet contains 0.46 wt. % calcium. Diet compositions and fatty acids distribution in triacylglycerols of diet fat are shown in S1 Table and Table 1, respectively. Body weight and food consumption were recorded weekly.

**Table 1:** Fatty acids content in the chow diet and fatty acids distribution in triacylglycerols of fats in animal feeds.

Fatty acids^1^^)^	Low *sn*-2 PA fat				Medium *sn*-2 PA fat				High *sn*-2 PA fat			
	mmol/100g diet	Total (mol%^4^^)^)	*sn*-2(mol%)	*sn*-1,3^3^^)^(mol%)	mmol/100g diet	Total (mol%)	*sn-*2(mol%)	*sn*-1,3(mol%)	mmol/100g diet	total (mol%)	*sn*-2(mol%)	*sn*-1,3(mol%)
C12:0	0.2	0.4	0.6	0.3	0.2	0.3	0.4	0.3	0.2	0.3	0.4	0.3
C14:0	0.8	1.5	1.0	1.7	0.8	1.4	1.8	1.2	0.8	1.4	2.3	0.9
C16:0	20.8	37.5	13.6	49.4	20.5	36.9	44.7	33.0	20.4	36.7	62.0	24.3
C18:0	1.9	3.4	6.2	2.1	1.8	3.3	6.5	1.7	1.8	3.2	6.5	1.5
C18:1*n*-9	23.7	42.7	56.7	35.7	25.8	46.0	36.3	51.6	26.7	48.1	23.6	60.4
C18:1-trans	0.1	0.2	0.2	0.2	0.2	0.2	0.2	0.3	0.2	0.3	0.2	0.4
C18:2*n*-6	7.2	13.0	20.8	9.1	5.3	9.6	8.8	10.0	4.5	8.2	3.5	10.5
C18:2-trans	0.2	0.4	0.6	0.2	0.2	0.3	0.7	0.1	0.2	0.4	1.1	0.1
C20:0	0.2	0.4	0.2	0.4	0.1	0.3	0.2	0.3	0.1	0.2	0.2	0.2
C16:0 esterified in *sn*-2 position^2^^)^(%)			12.1				40.4				56.3	

^1)^Fatty acids with lower concentration (<0.10%) are not listed above.

^2)^The percentage C16:0 esterified in sn-2 position is calculated by [(*sn*-2 C16:0 (mol %)) / (3×totalC16:0 (mol %)) ] × 100.

^3)^The percentage fatty acid esterified in *sn*-1, 3 position is calculated by *sn*-1, 3 (mol %) = [3 × total (mol %)—*sn*-2 (mol %)] /2.

^4)^Mol % indicates molar fraction percentage.

### Collection of samples

After feeding experiment, rats were individually placed in metabolism cages, where fresh fecal samples were collected separately for 3 days and subsequently stored at -20°C before analysis. During this time, the water and feeds were offered *ad libitum*. The food intake was determined for 3 days. After 3 days collection, all animals were anesthetized by intraperitoneal injection of pentobarbital sodium (60 mg/kg). The epididymal fat and perirenal fat tissues were obtained from the carcass, weighed and stored at -80°C.

### Total fecal fatty acids, fecal soap fatty acids and fecal calcium analysis

Fecal fatty acids and fecal soap fatty acids compositions were extracted and analyzed following the method reported by Quinlan et al. [22] with minor modification. Briefly, 500 mg of freeze-dried and powdered fecal samples was used for test. Neutral lipids were extracted from the sample by reflux with petroleum ether (boiling point, 30–60°C). The remaining sample was acidified by acetic acid to convert fatty acid soaps to free fatty acids (soap fatty acids), which were then extracted by reflux with petroleum ether (boiling point, 30–60°C). A known amount of internal standards C17:0 (Sigma-Aldrich Co Ltd, Shanghai, China) were added to two lipid extracts. And the non-soap fatty acids in the neutral lipids extract was separated by preparative TLC using the mobile phase n-hexane/diethyl ether/formic acid 70:30:1 (v/v/v) as developer. Fatty acid methyl esters were synthesized by use of boron trifluoride and methanol followed by GC analysis method [20]. Total fecal fatty acids expressed as the sum of non-soap fatty acid and soap fatty acids. Freeze-dried and powdered feces samples (100 mg) were used for total fecal calcium concentration analysis. The total fecal calcium concentration were determined by the ICP-MS method reported by Li et al. [23].

### Fecal DNA extraction

Bacterial DNA was extracted from pretreated fecal samples using commercial stool DNA extraction kit according (InviMag Stool DNA Kit/Kfml, STRATEC Molecular, Berlin, Germany). The extracted DNA was subsequently automated on the KingFisher Flex Magnetic Particle Processors (Thermo Scientific Inc., Bonn, Germany). The concentration of extracted DNA was assessed using a Nano-Drop 1000 spectrophotometer (Nano-Drop Technologies, Wilmington, DE). The extracted DNA samples were stored at—20°C for further analysis.

### PCR amplification and sequencing

To analyze the composition of microbiota community, universal forward primers 338F (5'- ACTCCTACGGGAGGCAGCA-3') and the reverse primer 806R (5’- GGACTACHVGGGTWTCTAAT-3') targeting the V3-V4 region of the 16S rRNA gene were chosen for the amplification and sequencing. The targeted 16S rRNA gene was PCR amplified in triplicate. Each PCR reaction was carried out in a 20 μL reaction mixtures containing 2 μL of 2.5 mMdNTPs, 4 μL of 5× FastPfu Buffer, 10 ng of DNA, 0.4 μL of each primer (5 μM) and 0.4 μLFastPfu Polymerase (TransGen, China).PCR conditions were as follows: denaturing step at 95°C for 3 min, following by 27 cycles of denaturing at 95°C for 30 s, annealing at 55°C for 30s and extension at 72°C for 45 s, followed by final annealing extension step for 10 min at 72°C. PCR products were visualized on a 2% agarose gel to assess the quality, and purified and quantified using Mini Elute PCR purification kit (AXYGEN) and ABI GeneAmp® 9700 system, respectively. Pyrosequencing was performed on a MiSeqIllumina platform at Majorbio Bio-pharm Technology Co., Ltd (Shanghai, China). The 16S rDNA sequences were deposited with the NCBI short reads archive data base under study accession number: PRJNA303100.

### Fecal SCFA content analysis

Approximately 3 g of fresh feces was added to 50 mL polypropylene vials and 10 mL Milli-Q deionised water added. The mixture was shaken for 1 min on a vortex mixer. The extraction was performed for 10 min using an ultrasonic cleaning bath (SK7200HP, Kudos Ultrasonic instrument co., Ltd, Shanghai, China) operating at 50 kHz frequency, following centrifugation for 10 min at 10 000 rpm.1mL of the supernatant was transferred to 10mLvolumetric flask, 100μL of 50 mmol H_2_SO_4_ was added, and made up to the volume (10 mL) with Milli-Q deionised water. The solution was filtered using a 0.45 μm PVDF filter. Standard solutions containing 1.0, 2.5, 5.0, 10.0, 20.0, 30.0, 40.0 mg/kg of acetic acid, propionic acid and butyric acid (Sigma-Aldrich Co Ltd, Shanghai, China), respectively, were used for calibration (R^2^>0.999). The concentration of SCFA was measured using a Metrohm ion chromatograph (850 Professional IC, Metrohm, Herisau, Swizerland) system, fitted with a Metrosep Organic Acids column (250 mm × 7.8 mm i.d., 5 μm particle size) and connected to a conductivity detector (Model 819, Metrohm, Herisau, Switzerland). The column oven was kept at 45°C. An isocratic elution was obtained with 0.5mmol/L H_2_SO_4_ as mobile phase at a flow-rate of 0.5mL/min. Samples were applied with a loop injector (20μL loop). A 50 mMLiCl solution was used as suppressor regenerant. The chromatograms were analyzed by MagIC Net software (Metrohm, Herisau, Switzerland).

### Bioinformatics analysis

Sequencing data were analyzed using the MOTHUR software (http://www.mothur.org/wiki/Main_Page). All the sequencing reads were denoised, and the low quality sequences, pyrosequencing errors and chimera were removed. Thereafter, high-quality reads were clustered into taxonomic units (OTUs) at 97% similarity level. The rarefraction curves, alpha diversity (within sample) indices (Chao1, ACE, Shannon and Simpson) and Good’s coverage analysis were performed using the MOTHUR software. Beta diversity (among samples) was analyzed by using principal coordinates analysis (PCoA).Venn diagrams were generated using Vennerable R package (http://r-forge.r-project.org/projects/vennerable). BLASTs of taxonomic classification down to the phylum and genus level were then performed using MOTHUR and the Bacterial SILVA106 database (http://www.arbsilva.de/documentation/background/release-106/).

### Statistical analysis

The results are presented as mean ± SD. Statistical analyses were performed by one-way ANOVA followed by post hoc tests (Tukey’s multiple comparison test). When data was not normally distributed, the Kruskal-Wallis test was used. All statistical analyses were carried out using GraphPad Prism Version 5.00 for Windows (GraphPad Software, San Diego, CA). *P*<0.05 was considered significant.

## Results and Discussion

### Food consumption, body mass gain and fat deposition

At first we looked at the effect of increasing concentrations of *sn*-2 PA fat on food consumption, body mass gain and fat deposition. During the 4-week feeding period, we did not see any significant differences in food intake, final body weight, body mass gain or fat deposition among the three groups (Table 2), indicating that the different levels of *sn*-2 PA fat supplementation do not affect growth of the animals.

**Table 2:** Feed intake and growth during the 4-week experimental period.

	Low *sn*-2 PA	Medium *sn*-2 PA	High *sn*-2 PA	*P*
Food intake (g/3d per rat)	16.9 ± 1.1	16.2 ± 0.6	17.7±1.2	0.28
Initial body weight (g)	98.6 ± 7.8	93.6 ± 6.6	94.1 ± 5.4	0.15
Finial body weight (g)	285 ± 39.3	280 ± 40.5	257 ± 52.5	0.26
Dry weight stool (g/3d)	4.29 ± 0.45	3.83 ± 0.35	3.96 ± 0.56	0.06
Epididymal fat (g)	1.86 ± 0.47	1.80 ± 0.35	1.69 ± 0.24	0.54
Perirenal fat (g)	1.80 ± 0.49	1.75 ± 0.30	1.61 ± 0.44	0.52

The results are the mean ± SD of 12 animals per group. *P* values were determined by one-way ANOVA followed by post hoc Tukey’s multiple comparison test.

### Effect of *sn-*2 PA levels on fecal fat and apparent fat absorption

In a next step we determined the influence of *sn*-2 PA on fat absorption. In the fecal matter, fatty acids majorly occur as soap or free acid, whereas triacylglycerol are absent and only small amounts of partial glycerides are observed in the stool [22]. Thus, in the present study, we focused on the compositions of fatty acid soaps and total fecal fatty acids in the feces. Since the food intake did not differ between the low, medium and high fat groups, the energy intake from fat was similar in each group (301kJ, 311kJ and 307kJ, respectively, Table 3).However, we saw a dose depended decrease of total fatty acids in the stool of animals fed increasing concentrations of *sn*-2 PA in the diet (*P*< 0.0001). The calculated loss of energy due to fecal fatty acid excretion was calculated to be 20.6 kJ, 10.7 kJ and 2.9 kJ in the low, medium and high fat groups (Table 3). Most of the fatty acids were secreted as soaps (86.2%, 80.0% and 73.3%,respectively) in all three groups. C16:0 was the predominant fatty acid present in the soap fatty acids in all groups. The concentration of C16:0 in the soap fatty acids of the low*sn*-2 PA diet group was significantly higher compared with the other two groups (*P* < 0.0001; Table 3), a 10-fold increase in comparison to the high *sn*-2 PA diet group (*P* < 0.0001) and a 2.5-fold increase in comparison to the medium*sn*-2 PA diet group (*P* < 0.0001). In addition, C18:1 and C18:2 excretion in the feces were lower in the group fed high *sn*-2 PA diet compared with low *sn*-2 PA diet and medium *sn*-2 PA diet groups (*P* < 0.0001).In brief, these data indicated that a greater level of *sn*-2 PA in the diet was associated with a greater absorption of dietary fatty acids and a reduction in fat energy loss, suggesting the nutritional benefits of *sn*-2 palmitate structured triacylglycerols.

**Table 3:** Three days fat intake, fecal fat excretion and absorption.

	Low *sn*-2 PA	Medium *sn*-2 PA	High *sn*-2 PA	*P*
Fat intake^1^^)^(mmol/3d)	9.2 ± 0.8	9.5 ± 0.7	9.4 ± 0.4	0.33
Energy intake^2^^)^ (kJ/3d)	301 ± 27	311 ± 24	307 ± 12	0.33
Total fecal fatty acid^3^^)^(mmol/3d)	2.1 ± 0.2^a^	1.0 ± 0.1^b^	0.3 ± 0.05^c^	< 0.0001
Energy loss^4^^)^ (kJ/3d)	20.6 ± 2.2^a^	10.7 ± 1.6^b^	2.9 ± 0.5^c^	< 0.0001
Non-soap fatty acids(mmol/3d)	0.26± 0.04^a^	0.15 ± 0.03^a^^,^^b^	0.1 ± 0.02^b^^,^^c^	< 0.0001
Soap fatty acids				
C16:0 (mmol/3d)	1.54± 0.17^a^	0.61 ± 0.1^b^	0.14 ± 0.05^c^	< 0.0001
C18:0 (mmol/3d)	0.13 ± 0.02^a^	0.08 ± 0.01^b^	0.04 ± 0.01^c^	< 0.0001
C18:1 (mmol/3d)	0.11 ± 0.1^a^	0.09 ± 0.01^b^	0.03 ± 0.01^c^	< 0.0001
C18:2 (mmol/3d)	0.02 ± 0.002^a^	0.01 ± 0.002^b^	0.002 ± 0.001^c^	< 0.0001
Total soap fatty acids^5^^)^ (mmol/3d)	1.81 ± 0.19^a^	0.80 ± 0.11^b^	0.22 ± 0.06^c^	< 0.0001

The results are the mean ± SD of 12 animals per group.

^a,b,c^ Mean values within a row with unlike superscript letters were significantly different (*P* < 0.05; Kruskal-Wallis test).

^1)^ Fat intake = fat intake (g)/ (885.4g/mol), 885.4 is triacylglycerol molecular weight.

^2)^ Energy intake (kJ/3d) is calculated based on fat intake. Energy: fat (37kJ/g).

^3)^ Total fecal fatty acid is the sum content of soap fatty acid and non-soap fatty acid in the feces.

^4)^ Energy loss is the fat energy losses in stool which calculated according to total fecal fatty acids. Energy: Fatty acid (37kJ/g).

^5)^ Total soap fatty acids is the sum of C16:0, C18:0, C18:1 and C18:2 content.

Our results are in good agreement with previous observations that long chain saturated fatty acids at *sn*-2 position of triacylglycerols were well absorbed compared with that located at *sn*-1, 3 positions [24]. During triacylglycerols hydrolysis by pancreatic lipase in the small intestine, fatty acids esterified at the *sn*-1 and *sn*-3 position are released as free fatty acids, whereas those esterified at the *sn*-2 position remain unhydrolyzed [25]. *Sn*-2 monoacylglycerols (containing saturated or unsaturated fatty acids) are well absorbed by the enterocytes and re-synthesized into new triacylglycerols, which are assembled into chylomicrons and secreted into the lymph system [26]. In contrast, the absorption of free fatty acids is determined by their physical and chemical properties. Unsaturated fatty acids (C18:1, C18:2, C18:3, etc.) and short chain saturated fatty acids are efficiently absorbed by enterocytes, since their melting points are lower than the intestinal temperature. This is the reason why C18:1 fatty acids, which are contained in the all three diets at an even higher concentration than C16:0 fatty acids, are only found in trace amounts in the stool. However, free saturated fatty acids with a long chain (C16:0, C18:0, etc.) can bind calcium in the gut and create non-soluble calcium soaps, which are subsequently excreted in the stool [27], thus the high amount of C16:0 fatty acids in the stool. This explains the greater absorption of C16:0 esterified at the *sn*-2 position than that of C16:0 esterified at the *sn*-1, 3 positions and the decrease of C16:0 in feces of the high *sn*-2 PA group. These results are also supported by previous findings [3] showing that a greater proportion of C16:0 at the *sn*-2 position resulted in a reduced fecal excretion of total soap fatty acids (mainly as C16:0). The authors used a rat model fed three types of fat with increased percentage (from 4.8% to 78.8%) of C16:0 esterified at the *sn*-2 position.However, in their report, the rats were only fed *sn*-2 PA fat for 3 days, while in the current study, a longer-term feeding duration of 4 weeks was carried out, showing that *sn*-2 PA fat can stably reduce total fatty acids C16:0 soaps in the feces of rats even for a longer-term.

### Effect of *sn*-2 PA levels on fecal calcium excretion and apparent calcium absorption

Since free fatty acids can bind calcium to build soaps, we also investigated calcium excretion in rats fed with different concentrations of *sn*-2 PA fat. The calcium intake in all groups were similar due to the near identical food intake, while total calcium content in the stool decreased with increasing *sn*-2 PA fat in the diet, from 3.58 mmol/3d in the low *sn*-2 PA fat group to 3.03 mmol/3d and 2.48 mmol/3d in the medium and high groups, respectively (*P* < 0.0001; Table 4).The apparent absorption rate significantly improved from 42.6%, to 53.8%, to 61.0% (*P* < 0.0001; Table 4) as the percentage of PA in the *sn*-2 position increased in the dietary fat. The calcium loss was probably, at least in part, the result of the formation of insoluble fatty acid soaps with calcium, which occurs when *sn*-1, 3 long chain saturated fatty acids were enzymatically liberated from the triacylglycerols in the presence of calcium. However, we did not discriminate between calcium in soaps and “non-soaps”, because of the experimental challenge due to the poor solubility of the soap complex in organic solvents [22] to prove this assumption. Nevertheless, in the present study, fecal calcium was used to estimate the calcium absorption in the rat fed different *sn*-2 PA diets as previously reported in other studies [17, 23] and our results indicate a positive correlation between dietary *sn*-2 PA level and calcium absorption. This observation is in agreement with the finding by previous reports [1, 28], in which the authors found that fecal calcium excretion of preterm infants fed high *sn*-2 PA formula was significantly lower compared with infants fed low *sn*-2 PA formula. In contrast, the study by Lien et al. [3] observed no differences in the fecal calcium content when the rats were fed with three different dosages of sn-2 PA fat for 3 days, although they showed that *sn*-2 PA fat reduced C16:0 soaps in the feces. The discrepancy might be due to the differences in the ration of PA to calcium in the diet. Lien et al. [3] used a diet with a ratio of PA: calcium of 0.6:1 (mol/mol), whereas the ratio of PA: calcium was 1.7:1(mol/mol) in this study.

**Table 4:** Three days calcium intake, fecal calcium excretion and apparent absorption.

Group	Calcium intake(mmol/3d)	Fecal calcium excretion(mmol/3d)	Apparent absorption^1^^)^(mmol/3d)	Apparent absorption rate^2^^)^ (%)
Low *sn*-2 PA	6.25 ± 0.56	3.58 ± 0.41^a^	2.65 ± 0.44^a^	42.6 ± 5.3^a^
Medium *sn*-2 PA	6.45 ± 0.50	3.03 ± 0.33^b^	3.43 ± 0.61^b^	53.8 ± 6.8^b^
High *sn*-2 PA	6.35 ± 0.24	2.48 ± 0.44^c^	3.88 ± 0.44^b^^,^^c^	61.0 ± 6.8^c^
*P*	0.54	< 0.0001	< 0.0001	< 0.0001

The results are the mean ± SD of 12 animals per group.

^a,b,c^ Mean values within a column with unlike superscript letters were significantly different (*P*< 0.05; ANOVA followed by post hoc Tukey’s multiple comparison test).

^1)^ Apparent absorption (mmol) = Calcium intake (mmol)–fecal Calcium excretion (mmol)

^2)^ Apparent absorption rate (%) = [Apparent absorption (mmol)/Intake (mmol)] ×100

### Effect of *sn*-2 PA levels on fecal microbiota community

For all 24 samples, a total of 763,566 reads with an average read length of 438 bp were obtained from Miseq. Each library contains 22,745 to 41,099 reads, with different phylogenetic OTUs ranging from 322 to 577. The microbial richness and alpha diversity indexes relative to each fecal sample is presented in Table 5. There was no significant difference in the fecal microbial richness or alpha diversity among the three *sn*-2 PA groups indicating that increasing the percentage of PA esterified at the *sn*-2 position in the dietary fat did not change species diversity on gut microbiota at 97% identity.

**Table 5:** Richness and diversity indexes relative to each fecal sample (OTU cutoff of 0.03).

Group	Reads	OTUs	Alpha diversity			
			ACE	Chao1	Shannon	Simpson
Low *sn*-2 PA	31462 ± 5022	485 ± 80	541.5 ± 78.8	546.5 ± 73.8	4.39 ± 0.47	0.034 ± 0.016
Medium *sn*-2 PA	33490 ± 3656	499 ± 43	556.0 ± 31.7	559.5 ± 33.4	4.42 ± 0.24	0.029 ± 0.008
High *sn*-2 PA	30494 ± 6201	486 ± 48	552.5 ± 42.0	557.5 ± 44.4	4.33 ± 0.48	0.039 ± 0.026
*P*	0.49	0.88	0.86	0.87	0.92	0.89

The results are the mean ± SD of 8 animals per group. *P* values were determined by one-way ANOVA followed by post hoc Tukey’s multiple comparison test.

**Fig 1:**
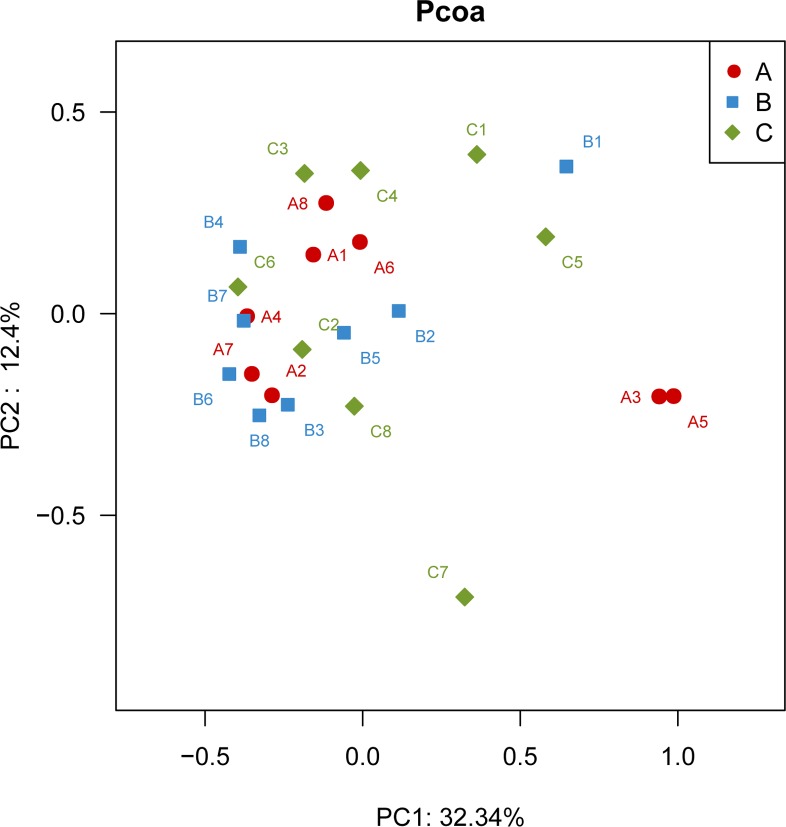
Principal coordinate analysis using Bray-Curtis distance (n = 8). The sample labels with letter and numeric A1-A8, B1-B8 and C1-C8 correspond to the eight random sample in the rats fed with low *sn*-2 PA fat, medium *sn*-2 PA fat and high *sn*-2 PA fat diets, respectively.

**Fig 2:**
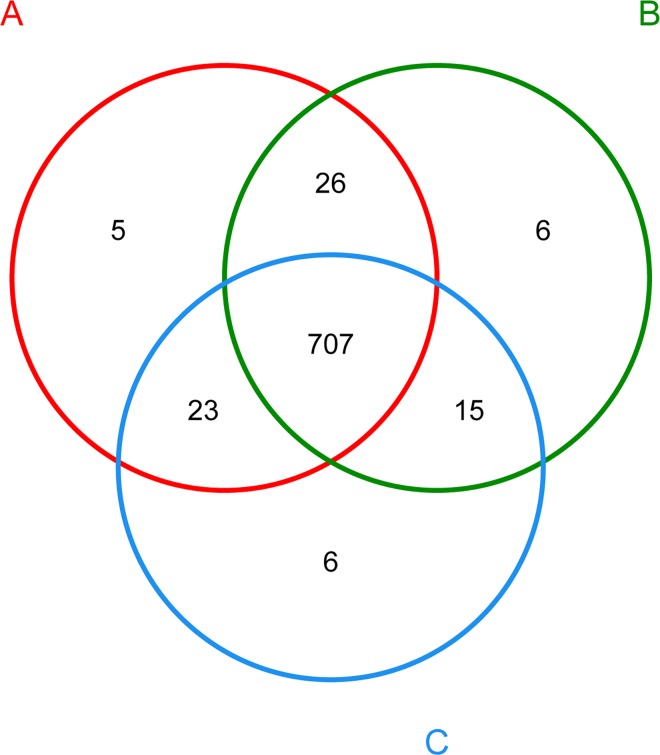
Venn diagram showing the unique and shared OTUs (3% distance level). The sample labels with letter A, B and C correspond to the rats fed low *sn*-2 PA fat, medium *sn*-2 PA fat and high *sn*-2 PA fat diets, respectively.

The principal coordinates analysis (PCoA) using Bray-Curtis distance matrix depicts the variance of different bacterial communities (Fig 1). The results showed that the first two factors, P1 and P2, could explain 32.3% and 12.4% variations, respectively. In addition, the species shared among these bacterial communities were determined by a Venn diagram to compare the relationships among these bacterial communities in detail. The results indicated that the low, medium and high *sn*-2 PA groups shared 707 OTUs, whereas only 5, 6 and 6 OTUs were unique to the respective groups (Fig 2). The PCoA and Venn diagram profiles of the microbiota communities derived from the rats suggested that the animals in the three treatment groups harbored similar fecal microbiota. These results demonstrated that different percentages of PA esterified at the *sn*-2 position in the dietary fat did not alter the fecal microbial community structure and diversity.

**Fig 3:**
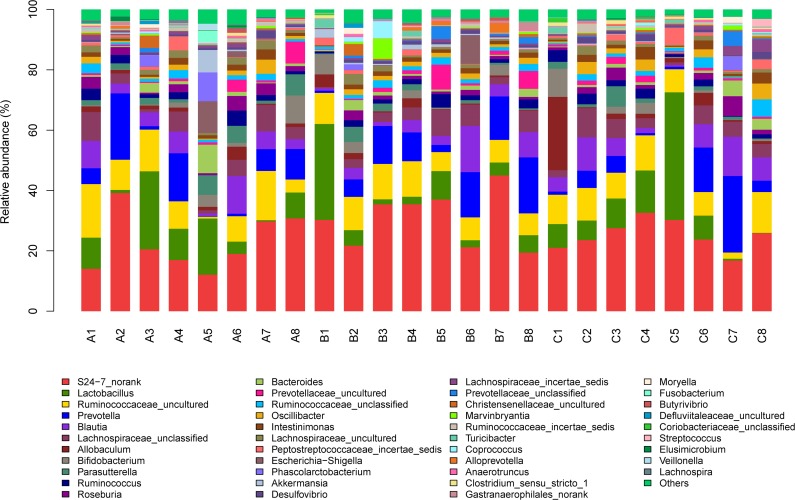
Fecal bacterial population at the genus level (n = 8). Relative abundance of different bacterial genus within the different communities. Sequences that could not be classified into any known group were assigned as ‘Unclassified bacteria’ and ‘No_Rank’. The ones with an abundance less than 1% in the phyla and genera were combined as “others”. The sample labels with letter and numeric A1-A8, B1-B8 and C1-C8 correspond to the eight random sample in the rats fed with low *sn*-2 PA fat, medium *sn*-2 PA fat and high *sn*-2 PA fat diets, respectively.

In addition, the effect of dietary *sn*-2 PA levels on taxonomic composition was investigated. A total of 9 major different phyla (data not shown) and42major different genera (Fig 3) were identified according to 16S rDNA gene sequencing data. Sequences that could not be classified into any known group were identified as no rank. At phylum level, *Firmicutes*, *Bacteroidetes* and *Proteobacteria* were the most important groups (data not shown), which together represent 95.2%, 95.3% and 96.8% on average of total reads in libraries of the low, medium and high dietary *sn*-2 PA groups, respectively. However, changes in *Firmicutes* (*P* = 0.10), *Bacteroidetes* (*P* = 0.16) and *Proteobacteria* (*P* = 0.37) among the three groups, did not reach statistical significance using the Kruskal-Wallis test. Taken together, these results suggested that different dietary *sn*-2 PA levels did not alter gut microbiota at phylum level.

At the genus level, the mean relative abundance of *Lactobacillus* was 9.91%, 7.88% and11.1%, respectively, among the three treatment groups, whereas the mean relative abundance of *Bifidobacterium* was 2.13%, 2.13% and 2.15%, respectively, among the three treatment groups, but did not reach statistical significance for both bacteria. This result did not agree with a previous observation [16] that term infants fed with high *sn*-2 PA fat diets had higher level of *Lactobacillus* and *Bifidobacterium* compared with those receiving low *sn*-2 PA diets. The discrepancy is probably due to the differences in hosts and their living conditions including diet [29].The total average relative abundance of short-chain fatty acid producing *Blautia* and *Allobaculum* was found to be higher in high *sn*-2 PA group than in the low, medium *sn*-2 PA groups (10.9%, 7.4% and 6.5%, respectively). Those two genera are believed to alleviate the host metabolic diseases and enhance the beneficial effects to the gut by producing short-chain fatty acids [30].In addition, the mean relative abundance of *Escherichia-Shigella*, one of opportunistic pathogens, was 1.68% and 1.26% in low and medium *sn*-2 PA groups, respectively. However, the opportunistic pathogen was not found in the high *sn*-2 PA group.

In previous studies [16, 17], culture-dependent or fluorescent in situ hybridization (FISH) were used to characterize the relationships between *sn-*2 PA level and gut microbiota. However, these techniques are limited in application due to several practical difficulties including the limited selectivity of the commonly used culture media, incapability to identify unknown species and reflect a limited view of the diversity and dynamics of the gut microbiota [31,32].Therefore, these studies only focused on the changes of known groups of bacteria during *sn*-2 PA structured triacylglycerols consumption, and there were no report on the overall effects of *sn*-2 PA structured triacylglycerols on the gut flora. Owing to the 16S rRNA high-throughput sequencing technology used in this study, we effectively reflect the changes of the entire profile of the gut microbiota community rather than focusing on only limited and known bacteria. We found that the whole microbiota community showed a remarkable similarity at phylum level and the difference at genera level were minimal in the three treatment groups.

### Effect of *sn*-2 PA levels on fecal SCFA content

SCFA are the end products of the microbial metabolism in the gut, with acetic acid, propionic acid and butyric acid being the major SCFA in the mammalian gut [33]. They have important metabolic functions and are crucial for intestinal health. Therefore, we investigated the effect of increasing concentrations of *sn*-2 PA fat on SCFA production. The total content of SCFA (sum of acetic acid, propionic acid and butyric acid) in the fecal matter was found to be highest in the high *sn*-2 PA fat fed group compared with the low *sn*-2 PA fat and medium *sn*-2 PA fat dietary groups (*P* < 0.0001; Table 6). Of these SCFA, the content of acetic acid and butyric acid were higher in the high *sn*-2 PA fat fed group compared with the low *sn*-2 PA fat and medium *sn*-2 PA fat dietary groups (*P* < 0.05).

**Table 6:** Fecal content of SCFA in rats (μmol/g).

	Low *sn*-2 PA	Medium *sn*-2 PA	High *sn*-2 PA	*P*
Acetic acid	22.1 ± 1.9^a^	23.5 ± 4.7^a^	30.2 ± 2.7^b^	< 0.0001
Propionic acid	5.5 ± 1.8	4.8 ± 0.7	5.9 ± 1.2	0.28
Butyric acid	2.3 ± 0.6^a^	2.6 ± 0.7^a^^,^^b^	3.3 ± 0.4^b^	0.01
Total SCFA^1)^	29.8 ± 2.8^a^	30.9 ± 4.6^a^	39.5 ± 3.1^b^	< 0.0001

The results are the mean ± SD of 12 animals per group.

^a,b^Mean values within a row with unlike superscript letters were significantly different (*P*< 0.05; ANOVA followed by post hoc Tukey’s multiple comparison test).

^1)^ Total SCFA is sum of acetic acid, propionic acid and butyric acid.

In the present study, SCFA levels were found to be altered by high *sn*-2 PA fat supplementation with higher content of acetic acid, butyric acid and total SCFA detected in the feces, even though all groups were fed a similar composition of diet except of the fat triacylglycerols structure. This can be explained, at least in part, by the higher abundance of bacteria responsible for SCFA production, like, which were found to be more abundant in the high *sn*-2 PA group.

In summary, the present study highlighted that total fat and calcium absorption were significantly improved by increasing the amount of PA esterified at the *sn*-2 position in the diet, which was probably due to the decrease in insoluble calcium-palmitate soaps. Furthermore, it is interesting to note that the total SCFA concentration was significantly elevated in the high *sn*-2 PA group. This observation was at least in part due to modification the abundance of gut microbiota composition (e.g. sum of *Blautia* and *Allobaculum*) which promoted SCFA production. In turn, the higher concentration of SCFA might promote calcium absorption by lowering pH in the intestinal and convert insoluble calcium soap to soluble calcium or calcium ions [34]. However, one caveat of our study might be that we did not consider a control group in which the rats were fed the standard basic diet for rats. In this experiment, low *sn*-2 PA fat (palm olein) was considered as the reference group, because the objective of this study was primarily focused on whether *sn*-2 PA fat could influence intestinal fatty acids absorption, calcium absorption, microbiota composition and SCFA concentrations. We used this group because palm olein is commonly used in infant formula. In contrast, the standard basic diet contains soybean oil rich in polyunsaturated fatty acids which is significantly different compared to the diets in the current study which mainly contained PA and oleic acid. Meanwhile, the design of this study was also chosen in previously studies [35, 36]. Despite this minor flaw we believe our work could be a springboard for studying in more detail the relationship between saturated fatty acids bound to the *sn*-2 position and fecal microbiota compositions or SCFA concentration.

## Conclusions

In conclusion, the present study demonstrated that a high *sn*-2 PA fat diet can effectively improve fatty acids and calcium absorption *in vivo*. This study also indicate that 16S rRNA high-throughput sequencing technology was helpful in investigating the overall effects of *sn*-2 PA fat on the composition of intestinal microbiota. Although *sn*-2 PA fat did not change the whole profile of the gut microbiota community, we saw an increase in SCFA producing bacteria genera with the high*sn*-2 PA fat diet which was accompanied by an increasing amount of SCFA in the feces. This observations suggest that the *sn*-structure of fat might play an important role in the modulation of gut SCFA and warrant future studies involving more *sn*-2 fatty acids with different chain length, degree of saturation and configurations.

## Supporting Information

S1 Table Diet composition. (DOCX)

## References

[1] Tomarelli RM, Meyer BJ, Weaber JR, Bernhart FW. Effect of Positional Distribution on the Absorption of the Fatty Acids of Human Milk and Infant Formulas. The Journal of Nutrition. 1968;95(4):583–90. . 5665659 10.1093/jn/95.4.583

[2] Wei W, Feng Y, Zhang X, Cao X, Feng F. Synthesis of structured lipid 1,3-dioleoyl-2-palmitoylglycerol in both solvent and solvent-free system. LWT—Food Science and Technology. 2015;60(2, Part 2):1187–94. 10.1016/j.lwt.2014.09.013

[3] Lien EL, Boyle FG, Yuhas R, Tomarelli RM, Quinlan P. The effect of triglyceride positional distribution on fatty acid absorption in rats. J Pediatr Gastroenterol Nutr. 1997;25(2):167–74. . 9252903 10.1097/00005176-199708000-00007

[4] Nelson SE, Frantz JA, Ziegler EE. Absorption of fat and calcium by infants fed a milk-based formula containing palm olein. Journal of the American College of Nutrition. 1998;17(4):327–32. . 9710840 10.1080/07315724.1998.10718770

[5] Wu H, Tremaroli V, Bäckhed F. Linking Microbiota to Human Diseases: A Systems Biology Perspective. Trends in Endocrinology & Metabolism. 2015;26(12):758–70. 10.1016/j.tem.2015.09.011 26555600

[6] Blaut M, Klaus S. Intestinal Microbiota and Obesity In: Joost H-G, editor. Appetite Control. Handbook of Experimental Pharmacology. 209: Springer Berlin Heidelberg; 2012 p. 251–73. 10.1007/978-3-642-24716-3_11 22249818

[7] Kau AL, Ahern PP, Griffin NW, Goodman AL, Gordon JI. Human nutrition, the gut microbiome, and immune system: envisioning the future. Nature. 2011;474(7351):327–36. 10.1038/nature10213 PMC3298082. 21677749 PMC3298082

[8] Qin J, Li Y, Cai Z, Li S, Zhu J, Zhang F, et al A metagenome-wide association study of gut microbiota in type 2 diabetes. Nature. 2012;490(7418):55–60. 10.1038/nature11450 23023125

[9] Belkaid Y, Hand T. Role of the Microbiota in Immunity and inflammation. Cell. 2014;157(1):121–41. 10.1016/j.cell.2014.03.011 PMC4056765. 24679531 PMC4056765

[10] Giongo A, Gano KA, Crabb DB, Mukherjee N, Novelo LL, Casella G, et al Toward defining the autoimmune microbiome for type 1 diabetes. The ISME journal. 2011;5(1):82–91. 10.1038/ismej.2010.92 PMC3105672. 20613793 PMC3105672

[11] de Wit N, Derrien M, Bosch-Vermeulen H, Oosterink E, Keshtkar S, Duval C, et al Saturated fat stimulates obesity and hepatic steatosis and affects gut microbiota composition by an enhanced overflow of dietary fat to the distal intestine: Am J Physiol Gastrointest Liver Physiol 2012 2012-09-01 00:00:00. G589-G99 p. 10.1152/ajpgi.00488.2011 22700822

[12] Devkota S, Wang Y, Musch MW, Leone V, Fehlner-Peach H, Nadimpalli A, et al Dietary-fat-induced taurocholic acid promotes pathobiont expansion and colitis in Il10-/- mice. Nature. 2012;487(7405):104–8. 10.1038/nature11225 22722865 PMC3393783

[13] Fava F, Gitau R, Griffin BA, Gibson GR, Tuohy KM, Lovegrove JA. The type and quantity of dietary fat and carbohydrate alter faecal microbiome and short-chain fatty acid excretion in a metabolic syndrome 'at-risk' population. International journal of obesity (2005). 2013; 37(2):216–23. 10.1038/ijo.2012.33 . 22410962

[14] Patterson E, O' Doherty RM, Murphy EF, Wall R, O' Sullivan O, Nilaweera K, et al Impact of dietary fatty acids on metabolic activity and host intestinal microbiota composition in C57BL/6J mice. Br J Nutr. 2014;111(11):1905–17. 10.1017/S0007114514000117 24555449

[15] Marques TM, Wall R, O'Sullivan O, Fitzgerald GF, Shanahan F, Quigley EM, et al Dietary trans-10, cis-12-conjugated linoleic acid alters fatty acid metabolism and microbiota composition in mice. Br J Nutr.2015; 113(5):728–38. 10.1017/S0007114514004206 25697178

[16] Yaron S, Shachar D, Abramas L, Riskin A, Bader D, Litmanovitz I, et al Effect of High beta-Palmitate Content in Infant Formula on the Intestinal Microbiota of Term Infants. J Pediatr Gastroenterol Nutr. 2013;56(4):376–81. 10.1097/MPG.0b013e31827e1ee2 . 23201699

[17] Yao M, Lien EL, Capeding MR, Fitzgerald M, Ramanujam K, Yuhas R, et al Effects of Term Infant Formulas Containing High sn-2 Palmitate With and Without Oligofructose on Stool Composition, Stool Characteristics, and Bifidogenicity. J Pediatr Gastroenterol Nutr. 2014;59(4):440–8. 10.1097/mpg.0000000000000443 . 24840511 PMC4222706

[18] Zoetendal EG, Rajilić-Stojanović M, de Vos WM. High-throughput diversity and functionality analysis of the gastrointestinal tract microbiota. Gut. 2008;57(11):1605–15. 10.1136/gut.2007.133603 18941009

[19] Xu X, Balchen S, Høy CE, Adler-Nissen J. Production of specific-structured lipids by enzymatic interesterification in a pilot continuous enzyme bed reactor. J Am Oil Chem Soc. 1998;75(11):1573–9. 10.1007/s11746-998-0096-6

[20] IUPAC, Standard Methods for the Analysis of Oils, Fats and Derivatives, Pergamon Press Publications, 6thedition. (1979); IUPAC Method 2.301 and 2.302.

[21] AOCS, Official Methods and Recommended Practices of the AOCS, 6th edition (2013); AOCS Official Method Ch 3–91.

[22] Quinlan PT, Lockton S, Irwin J, Lucas AL. The relationship between stool hardness and stool composition in breast- and formula-fed infants. J Pediatr Gastroenterol Nutr. 1995;20(1):81–90. . 7884622 10.1097/00005176-199501000-00014

[23] Li Y, Mu H, Andersen JET, Xu X, Meyer O, Ørngreen A. New human milk fat substitutes from butterfat to improve fat absorption. Food Research International. 2010;43(3):739–44. 10.1016/j.foodres.2009.11.006

[24] Brink EJ, Haddeman E, de Fouw NJ, Weststrate JA. Positional Distribution of Stearic Acid and Oleic Acid in a Triacylglycerol and Dietary Calcium Concentration Determines the Apparent Absorption of these Fatty Acids in Rats. The Journal of Nutrition. 1995;125(9):2379–87. 7666256 10.1093/jn/125.9.2379

[25] Wang TY, Liu M, Portincasa P, Wang DQH. New insights into the molecular mechanism of intestinal fatty acid absorption. European Journal of Clinical Investigation. 2013;43(11):1203–23. 10.1111/eci.12161 24102389 PMC3996833

[26] Small DM. The effects of glyceride structure on absorption and metabolism. Annu Rev Nutr. 1991; 11:413–34. 10.1146/annurev.nu.11.070191.002213 . 1892708

[27] Lien EL. The role of fatty acid composition and positional distribution in fat absorption in infants. The Journal of pediatrics. 1994;125(5 Pt 2):S62–8. . 7965455 10.1016/s0022-3476(06)80738-9

[28] Lucas A, Quinlan P, Abrams S, Ryan S, Meah S, Lucas P. Randomised controlled trial of a synthetic triglyceride milk formula for preterm infants. Archives of Disease in Childhood Fetal and Neonatal Edition. 1997;77(3):F178–F84. PMC1720718. 9462186 10.1136/fn.77.3.f178 PMC1720718

[29] Xiao L, Feng Q, Liang S, Sonne SB, Xia Z, Qiu X, et al A catalog of the mouse gut metagenome. 2015;33(10):1103–8. 10.1038/nbt.3353 . 26414350

[30] Zhang X, Zhao Y, Zhang M, Pang X, Xu J, Kang C, et al Structural changes of gut microbiota during berberine-mediated prevention of obesity and insulin resistance in high-fat diet-fed rats. PLoS One. 2012;7(8):e42529 10.1371/journal.pone.0042529 . 22880019 PMC3411811

[31] Nelson GM, George SE. Comparison of media for selection and enumeration of mouse fecal flora populations. Journal of Microbiological Methods. 1995; 22(3):293–300. 10.1016/0167-7012(95)00004-5

[32] Fraher MH, O'Toole PW, Quigley EMM. Techniques used to characterize the gut microbiota: a guide for the clinician. Nat Rev Gastroenterol Hepatol. 2012;9(6):312–22. 10.1038/nrgastro.2012.44 22450307

[33] Koh A, De Vadder F, Kovatcheva-Datchary P, Bäckhed F. From Dietary Fiber to Host Physiology: Short-Chain Fatty Acids as Key Bacterial Metabolites. Cell. 165(6):1332–45. 10.1016/j.cell.2016.05.041 27259147

[34] Ohta A, Ohtuki M, Takizawa T, Inaba H, Adachi T, Kimura S. Effects of fructooligosaccharides on the absorption of magnesium and calcium by cecectomized rats. Int J VitamNutr Res, 1994; 64:316–323. . 7883472

[35] Carta G, Murru E, Lisai S, Sirigu A, Piras A, Collu M, Batetta B, Gambelli L, Banni S. Dietary triacylglycerols with palmitic acid in the sn-2 position modulate levels of N-acylethanolamides in rat tissues. PLoS One. 2015; 10(3):e0120424 10.1371/journal.pone.0120424 . 25775474 PMC4361611

[36] Renaud SC, Ruf JC, Petithory D. The positional distribution of fatty acids in palm oil and lard influences their biologic effects in rats. J. Nutr. 1995; 125(2):229–237.. 7861250 10.1093/jn/125.2.229

