# Supplementary material for: Characterisation of Fecal Soap Fatty Acids, Calcium Contents, Bacterial Community and Short-Chain Fatty Acids in Sprague Dawley Rats Fed with Different sn-2 Palmitic Triacylglycerols Diets
Source: PLoS One. 2016 Oct 26;11(10):e0164894. doi: 10.1371/journal.pone.0164894 (PMC5082633; doi:10.1371/journal.pone.0164894)
Supplement: S1 Table — (DOCX) [file pone.0164894.s001.docx]

**S1 Table. Diet composition**

|  | **Low *sn*-2 PA** | | **Medium *sn*-2 PA** | | **High *sn*-2 PA** | |
| --- | --- | --- | --- | --- | --- | --- |
|  | g% | *kcal%* | g% | *kcal%* | g% | *kcal%* |
| Protein | 15.5 | *14* | 15.5 | *14* | 15.5 | *14* |
| Carbohydrate | 62.0 | *56* | 62.0 | *56* | 62.0 | *56* |
| Fat | 15.0 | *30* | 15.0 | *30* | 15.0 | *30* |
| Total |  | *100.0* |  | *100.0* |  | *100.0* |
| kcal/g | 4.45 |  | 4.45 |  | 4.45 |  |
| Ingredient | g | *kcal* | g | *kcal* | g | *kcal* |
| Casein | 170 | *680* | 170 | *680* | 170 | *680* |
| L-Cystine | 2.55 | *10.2* | 2.55 | *10.2* | 2.55 | *10.2* |
| Corn Starch | 297.93 | *1191.7* | 297.93 | *1191.7* | 297.93 | *1191.7* |
| Sucrose | 353.18 | *1412.7* | 353.18 | *1412.7* | 353.18 | *1412.7* |
| maltodextrin 10 | 29.75 | *119* | 29.75 | *119* | 29.75 | *119* |
| Cellulose,BW 200 | 42.5 | *0* | 42.5 | *0* | 42.5 | *0* |
| Low *sn*-2 PA fat | 166.66 | *1499.9* |  |  |  |  |
| Medium *sn*-2 PA fat |  |  | 166.66 | *1499.9* |  |  |
| High *sn*-2 PA fat |  |  |  |  | 166.66 | *1499.9* |
| Mineral Mix S10026 | 8.5 | *0* | 8.5 | *0* | 8.5 | *0* |
| DiCalcium Phosphate | 11.05 | *0* | 11.05 | *0* | 11.05 | *0* |
| Calcium Carbonate | 4.68 | *0* | 4.68 | *0* | 4.68 | *0* |
| Potassium Citrate,1H2O | 14.03 | *0* | 14.03 | *0* | 14.03 | *0* |
| Vitamin Mix V10001 | 8.5 | *34* | 8.5 | *34* | 8.5 | *34* |
| Choline Bitartrate | 1.7 | *0* | 1.7 | *0* | 1.7 | *0* |
| FD&C Red Dye #40 | 0.02125 | *0* | 0.02125 | *0* | 0.02125 | *0* |
| FD&C Blue Dye #1 | 0.02125 | *0* | 0.02125 | *0* | 0.02125 | *0* |
| **Total** | **1111.05** | ***4948*** | **1111.05** | ***4948*** | **1111.05** | ***4948*** |
